# Debate: The concept of culture has outlived its usefulness for psychiatry

**DOI:** 10.1192/bjb.2017.15

**Published:** 2018-04

**Authors:** Mohammed A. Rashed, Rachel Bingham, Norman A. Poole, Abdi Sanati, Werdie van Staden

**Affiliations:** 1 Birkbeck, University of London, UK; 2 King's College London, UK; 3 Freedom from Torture, London, UK; 4 South West London & St George's Mental Health NHS Trust, UK; 5 East London National Health Service Foundation Trust, UK; 6 University of Pretoria, South Africa

## Abstract

**Summary:**

This paper presents a debate in which the authors participated at the World Psychiatric Association conference in Cape Town, South Africa in November 2016. Professor van Staden acted as chair and here, as at the debate, provides a rationale for debating a topic that many of those involved in mental health believe to be decided. The discussion that ensued demonstrated, however, that while the arguments have moved on they have not ceased. Who won? Well that depends how you look at it. A few in the audience shifted position towards the motion but the majority remained opposed. What do you think?

**Declaration of interest:**

None.

## Introduction: Werdie van Staden

‘Preposterous’ or ‘high time’ may be the respective responses of antagonists and protagonists to the claim that the concept of culture has outlived its usefulness for psychiatry. Philosophical rigour, however, requires much more than an exclamation of sentiment. Such rigour is probably demonstrated best by articulating an argument that goes against one's own sentiment, thereby unpacking and clarifying the issues at stake. This article follows suit, thus not committing our contributors beyond the remits of this article – an article that is about the ‘what’ (i.e. the content) as well as the ‘how’ (i.e. argument).

The topic is patently relevant considering the provisions for culture by the DSM-5,^1^ specifically through cultural concepts of distress, the cultural formulation and the cultural formulation interview. In addition, connections between culture and mental health may variously and profoundly be found in the way culture affects the various mental disorders in their expression, experience, interpretation, course and outcome. One may assert that culture influences individual resilience, coping mechanisms and social response, which are all crucial for recovery from mental health problems. Anthropologically, one may assert that mental health practice involves the meeting of at least two cultural worlds: the clinician's and the patient's.^2^

A scholarly debate provides for consideration, if of good logical pedigree, of the differences as well as the similarities shared by both sides of the debate. As an introduction to the arguments of our pairs of protagonists and antagonists, two shared features are highlighted: ‘culture’ necessarily involves *practice* and *values*.

The first protagonist, Mohammed Rashed, raises the issue of the conceptual relation between cultural group identity and culture as a dynamic set of shared practices. Taking the latter rather than the former as constitutive of culture, cultural group identity is shown to be a conceptual derivative of shared practice rather than the (commonly assumed) converse. The premise also underpins the antagonist argument of Abdi Sanati below. Following Wittgenstein's private language argument, he argues that without culture there cannot be language, and, hence, no psychiatry.

The premise that culture is constituted by shared practice thus features on both sides of the debate, with conceptual implications that are beyond the scope of this article. One example, nonetheless, may serve to enlighten their arguments. This premise unmasks the mistaken assumption that culture is necessarily confined to a geographical location (as does the DSM-5).^2^ Examples of the contrary are found in shared practice: the internet culture, the renaissance culture, a scientific culture, a 21st century culture, a ‘Googling’ culture, etc.

An emphasis on values is shared by the arguments of the protagonist, Norman Poole, and the antagonist, Rachel Bingham. The inevitable cultural origins of values are underscored in Bingham's argument, whereas Poole is concerned by the alienating and exoticising consequences of ascribing mental disorder to cultural values.

Space here does not allow for rebuttal, but the scene is set, no doubt, for further discussion.

## For the motion, Mohammed A. Rashed

I am going to argue for this motion: I believe that the concept of culture has outlived its usefulness for psychiatry. I believe so not because I am a defender of a reductive, biological psychiatry – I am not – but because among the two definitions of culture that are, in my view, of relevance to mental health, the first is so general so as to dissolve into the concept of meaning, and the second is so unjustifiably homogenising as to require that we explore the patient's beliefs and values without the assumption of a cultural group.

Culture has many definitions; I list four.

1 Culture in the sense of activity: the tending of natural growth; to cultivate the land, to breed animals, and to await the growth of bacteria in a Petri dish.^3^ This meaning of culture is irrelevant to our debate.
2 Culture also in the sense of activity, but instead of cultivating vegetables and bacteria, we cultivate our intellectual capacities and create civilisation. This meaning of culture lives today in the ‘culture’ section of newspapers.^4^ This meaning of culture, as well, is not relevant here.
3 Culture as a noun, denoting groups of people united by shared beliefs and practices; for example, Maori culture, Muslim culture, and so on.^5^ This meaning of culture is relevant.
4 Finally, culture as socially acquired and shared symbols, meanings and significances that structure experience, behaviour, interpretation and social interaction; culture ‘orients people in their ways of feeling, thinking, and being in the world’ (Jenkins & Barrett 2004,^6^ p. 5; see Rashed 2013,^4^ p. 4). This concept of culture is analytic in the sense that its introduction enables researchers and theoreticians to account for the specific nature of, and the differences among, various social phenomena and people's subjective reports of their experiences. For example, a prolonged feeling of sadness can be explained by one person as the effect of a neurochemical imbalance, by another as the effect of malevolent spirits, and by another as a test of one's faith: these differences can be accounted for through the concept of culture.

Having identified two meanings of culture that are relevant to this motion, I want to show why we should abandon the term culture. Consider the final definition: culture as socially acquired meanings and significances. Here we are talking about interpretation and giving meaning to our experiences, to the experiences of others, to social events and to behaviour. For example, in many societies, there exists the phenomenon of the evil eye.^2^ This is an innate capacity to do unintentional harm through a direct look when encountering abundance or beauty in situations that evoke genuine admiration and appreciation. It may affect animals, plants, material possessions and human beings. People for whom this notion is relevant regularly explain misfortune – for example, if a tree dies or a well dries – by saying that a person who visited recently had given a ‘bad eye’. For other people, however, seeing a friend's new car is an occasion to say something like ‘congratulations’ and to ask go for a drive, without worry that one might have, inadvertently, given the bad eye.

Now, both approaches to such a social encounter involve an interpretation and a related response, and this applies to all social encounters irrespective of where the involved individuals come from. But then what part does the term ‘culture’ play here? If everything is culture, in the sense that everything is subject to interpretation, then it would seem that we just need to attend to that without having to invoke an overarching thing called ‘culture’. It could be objected that the term culture is helpful because it allows us to identify a certain set of interpretations that occur together regularly, and to give that a name. This brings us to the third definition of culture I mentioned earlier: culture as a noun denoting groups. So, for example, the benefit of using the term Maori culture is that we can refer to a set of interpretations and practices that Maoris do: a shorthand to refer to a whole group and ascribe to them certain beliefs and practices. This, however, is problematic. The idea that we can demarcate a group of people that believe this or that and do such and such has been debunked as an anthropological fiction belonging to the European missionaries and adventurers of the first half of the previous century. It downplays individual agency in favour of some homogenous thing called ‘those people's culture’.^3^ Consider, again, the evil eye, a phenomenon which I studied in Egypt. No two people agreed on what it meant, on its importance, on the extent to which it is a genuine problem, or on the situations in which it can be harmful. In fact, the description I gave earlier is quite partial; people appropriated the notion and made it their own, and they had a unique sense of what it is and whether or not it is relevant to them.

The notion of a cultural group may be useful politically, but it's not useful for psychiatry. The clinical encounter must always involve a serious inquiry into the person's beliefs and values, and this has to occur every time irrespective of where the clinician and the patient come from; that is, irrespective of their presumed ‘culture’. In fact, to continue to use the term culture to refer to a group can be detrimental in that it may make the clinician think that he or she understands the patient – that the patient is ‘Muslim’ or ‘Maori’ or ‘Irish’ – when that understanding may be no more than a stereotype and hence a further obstacle to engaging with the other person's worldview. To recap, the concept of culture is no longer useful for psychiatry; if we understand culture as meanings and significances, then we need to stay with those notions, the term ‘culture’ playing no useful part. If we understand culture as referring to bounded groups, we would be holding a discredited view of culture, and a view that may prevent genuine engagement between clinicians and patients.

## Against the motion, Rachel Bingham

A central conceptual struggle in psychiatry is the question of how to define mental disorder, and thereby delineate the proper boundaries of psychiatric practice. The concept of culture may arise in various ways; for example, in drawing a distinction between religious experience and psychopathology,^7^ or between social deviance and mental disorder.^8^ Here, I will argue that the concept of culture is more useful than ever. My argument, briefly, is that the boundary between mental health and ill health is defined by values, and values arise from culture; therefore, mental health and psychiatric practice cannot be understood without attention to culture. Developing this, I suggest that not only do values depend on culture, but values and culture overlap, or partly constitute each other. Accordingly, to say something about a person's culture is to say something about her values, and vice versa. If so, the concept of culture should be considered vital to values-based practice.^9^

The concept of culture is used here to refer to socially acquired meanings that shape our experiences, our understandings of our experiences, the behaviours of others and social events more broadly. These shared meanings may influence not only when I believe myself to be unwell and what response I consider is required, but may also shape my experiences, actually contributing to whether or not I feel unwell or in need of help in the given circumstances.^7^ It is important not to overstate the homogeneity of cultural meanings within a group. I may not endorse all supposedly shared beliefs and values of all contexts I inhabit, and it will be difficult to make predictions about my beliefs and values by surveying the community in which I live. Nevertheless, I will be affected by others’ beliefs and values – whether I endorse them, unquestioningly go along with them, or even use my rejection of the beliefs and values of my community as a springboard from which to find my own way.

Values are integral to the concept of mental disorder, just as they are to the concept of culture. Attempts to define mental disorder in purely objective, factual terms have not been successful.^10^ Distinctions between mental disorder and mental health are irreducibly normative. Facts alone cannot tell us which statistically extreme deviations, such as genius or avarice, are pathological. Neither can facts alone tell us what is functional or dysfunctional, as functions themselves are relative to some valued outcome or purpose. Even if the neural correlates were described for every item in a list of diagnosable mental disorders, we would rely on values to decide the contents of that list. Otherwise, we would merely have a list of states involving changes in the brain, including, perhaps, love or criminality, with no further directions as to which are the proper business of psychiatry.^11^

Values, then, are essential to the concept of mental disorder. And values, as discussed, are not only influenced by culture, but contribute to culture, and vice versa. But if so, why not abandon culture altogether, as redundant, and focus only on what people value? Yet, to focus only on values and not their context does not serve psychiatry well. Whether values, meanings and interpretations are shared or not may influence whether a particular experience is interpreted as illness, or as some other unusual or distressing but essentially normal state. For example, whether experiences such as fear or hearing voices are shared and understood contributes to whether an individual is considered to be gifted, possessed, well or unwell. In other words, the values and beliefs of our community may partly constitute our experience of illness. The extent to which these meanings are shared may even determine whether or not a diagnosis is made.

In conclusion, among many uses of the concept of culture, one important feature is to describe something other than scientific facts: something essentially about values. Yet, psychiatry cannot focus only on values, abandoning culture, because part of both the experience and the diagnosis of mental health problems depends on the context – the shared beliefs and meanings – in which values arise and develop. Accordingly we need to see the practice of psychiatry as part of a wider context, as happening in a time and place that influence how it proceeds. We need to see our clients, patients, colleagues and ourselves as coming from contexts that influence not only whether we experience distressing or disabling symptoms, but also whether these symptoms are interpreted as illness. Unless we can say this is really being done well, without the need for further theoretical work, we cannot dispense with the concept of culture.

## For the motion, Norman A. Poole

As a neuropsychiatrically inclined psychiatrist, it might be assumed that my reservations stem from a preference for biological explanations in psychiatry. While there is something to be said for the role of neuroimaging and biological investigations in psychiatry – for example, the measurement of hippocampal volume and cerebrospinal fluid beta-amyloid concentration in early stage Alzheimer's disease – they do not satisfactorily address the motion, which, let us recall, is that culture has outlived its usefulness for psychiatry. This is not to argue that the focus on culture has hindered psychiatry. Indeed, the transcultural psychiatrists have, among others, helped us to see that biological reductionism is simplistic and wrong-headed. The diagnosis of mental disorder is at the normative level,^12^ just as the culturally orientated psychiatrists claimed. Furthermore, transcultural psychiatry has shown that what is deemed pathological in one culture need not be viewed as such within another. Demon possession is considered abnormal in the West, but this does not generalise to all other situations. What is left to discuss? It appears I am at one with my opponents.

My reservation is that cross-cultural psychiatry prioritises difference at the expense of universality, thereby exoticising mental disorder and potentially alienating patients further. Those with so-called culture-bound syndromes appear in the literature like new species of tropical bird for the reader to gape and wonder at. Worse still is the tendency, familiar to anyone who has worked in areas of diversity, for clinicians with a smattering of mandatory ‘cross-cultural training’ to dismiss unusual behaviour as ‘cultural’. I've heard this applied to new-onset domestic violence and social withdrawal; cases of frontal temporal dementia and schizophrenia, respectively, as it turned out.

Instead, I wish to present the view that what goes awry at the level of norms and values is more universal than the transcultural psychiatrists have supposed. Consider Pascal Boyer's notion of a folk psychiatry, which is parasitical on what is called intuitive psychology.^13^ That is, the tendency to understand one's own and others' behaviour through appeal to unobservable mental states such as beliefs, desires and emotions, including their relation to one another. Most of this is done at a level beneath conscious awareness; we become aware only of the outcome. And our intuitive expectations of one another are composed of domain-specific abilities rather than this being a general process. While there are differences in explicit psychological models around the world, the evidence from developmental psychology is that intuitive abilities are universal; the best-known being theory of mind, which occurs in all cultures studied to date.^14^ Other tenets of intuitive psychology include: mental states somehow represent or map the world as it is; behaviour is internally generated; memory is a store of past experience; communication follows tacit but constraining programmatic principles, with each party endeavouring to ensure the other's ongoing comprehension; a largely unconscious reading of others' subtle emotional cues; and so on. That these develop in infancy implies they are more universal than local. For instance, babies preferentially attend to objects that appear to interact with one another, such as the Heider and Simmel animation involving an ‘aggressive’ triangle and ‘fearful’ circle (https://www.youtube.com/watch?v=VTNmLt7QX8E). No one, with the exception of those with autism,^15^ has any trouble in attributing internal mental states to these shapes. Indeed, feelings of pity for the circle are frequently evoked.

The idea is that mental disorder is implied when behaviour, including verbal behaviour, contravenes one or more of our tacit expectations. People with schizophrenia exhibit disordered thought and speech that fails to follow the tacit rules of checking, repair, reducing ambiguity and so forth, which are apparent to carers and fill the psychopathology textbooks. It is important to note that this failure to meet the expectations of intuitive psychology are not mere violations of social norms – it is possible to behave in a socially unacceptable manner without there being a corresponding difficulty with its understandability. Repeat offenders are socially sanctioned, but few of us have trouble attributing a motive to their crimes. Intuitive psychology also seems to help sort the classic cross-cultural psychiatric cases, without recourse to culture. The belief that one is possessed by demons does not in the West seem to map or represent the world accurately. There are, however, other accepted means for the acquisition of belief; beliefs can also arise from the testimony of others.^16^ The belief that one is possessed by demons is accepted in some cultures, because the belief is acquired from authority, i.e. the rest of the group. This removes culture, because it is a fault with the mechanism of belief acquisition that triggers suspicion of dysfunction rather than the more general notion of a social norm being contravened.^17^

To conclude, culture has outlived its usefulness for psychiatry because it has misconceived the level at which things go wrong in mental disorder. Those with mental disorder are not identified merely for social deviancy but because some aspect of their behaviour fails to meet the intuitive and universally held psychological expectations of others. By focusing at this level, we are better able to appreciate what unites us, both in sickness and in health.

## Against the motion, Abdi Sanati

In what follows, I aim to show that culture is inseparable from psychiatry, and that, in fact, psychiatry cannot be practised, or conceived of, without culture. One can think of different ways to link psychiatry and culture. One of the most basic ones is through language. First, let us focus on the relationship between language and psychiatry. One of the important, and in my opinion essential, elements of psychiatry and its practice is psychopathology. It provides the framework within which we define signs and symptoms of mental disorders and communicate them to others. And language plays a necessary part in this discipline. From an ontological point of view, I find it hard to imagine the existence of delusions, verbal hallucinations and obsessional thoughts without language. From an epistemological point of view, to say any enquiry about human emotions is impoverished without use of language is an understatement. Even describing purely behavioural signs needs a language.

Now, I shall consider the relationship between language and culture. Culture and society are inseparable. One of the integral elements in every society is communication. Language is one of the most complex means of communication and has enabled human society to achieve immense complexity. The increase in the complexity of language contributed to the increase in the complexity of the culture. The increase in complexities of culture, in turn, feed back to make language more complex. There are many other factors operating in this process; for instance, I cannot deny the impact of technology on both culture and language. However, there is a definite link between culture and language. One can argue that while there is a definite association between culture and language, this association is merely a contingent one and it does not necessarily have to be the case. In the next step, I shall argue that the association is indeed necessary; that is, without culture we would not have language. Here, I rely on the work of Ludwig Wittgenstein, especially his private language argument. Wittgenstein explores whether there is a possibility of existence of a language which is logically private; that is, it could be understandable only by one person.^18^ To clarify, it is not the possibility of a language that someone like me can develop, which can be deciphered, but the possibility of a language by someone who has been separate from others since birth: a born Robinson Crusoe. This is different from development of a new language by someone who already is a language speaker. That person is already in possession of language skills, and the new language would follow accepted rules. The Crusoe-type person in question does not have any awareness of the rules of language and has to develop them from scratch. In other words, this language is developed *de novo* in an individual who has never been part of a community/culture. Wittgenstein concludes that ‘a language in principle unintelligible to anyone but its originating user is impossible. The reason for this is that such a so-called language would, necessarily, be unintelligible to its supposed originator too, for he would be unable to establish meanings for its putative signs’.^19^ Why would he be unable to do so? The answer lies in Wittgenstein's description of language. According to Wittgenstein, to understand a word is not to have a mental process signifying it. It is knowing *how* to use the word. In other words, it is to know how to follow the rules of using the words in different linguistic activities such as questioning, asserting, joking, demanding, etc. Language is a rule-governed activity. And to follow a rule one needs public criteria, i.e. something outside oneself to objectively confirm that the rule is followed. By objective, Wittgenstein does not mean that the rules are in some way independent of our practice, something he asserted in his earlier philosophy, but that what constitutes a rule is our collective use of it. Rule-following is a general practice established by agreement, custom and training.^20^ He argues that the concept of rule presupposes a custom. It is a cultural phenomenon. It cannot be imagined to happen individually, independent of ‘historical groups of individuals who are bound together into a community by a shared set of complex, language-involving practices’.^21^ There is a vital connection between language and the complex set of practices and activities that binds a community together. Language is interwoven into the activities of the people and is fundamentally cultural in nature. In other words, without culture there cannot be language and, hence, no psychiatry.

## About the authors

**Mohammed A. Rashed** is at the Department of Philosophy, Birkbeck, University of London, and the Department of Philosophy, King's College London, UK. **Rachel Bingham** is at Freedom from Torture, London, UK. **Norman A. Poole** is at the Department of Philosophy, King's College London, and South West London and St George's Mental Health National Health Service Trust, UK. **Abdi Sanati** is at the East London National Health Service Foundation Trust, UK. **Werdie van Staden** is at the Centre for Ethics and Philosophy of Health Sciences, Faculty of Health Sciences, University of Pretoria, South Africa.
